# Quality Assessment of TPB-Based Questionnaires: A Systematic Review

**DOI:** 10.1371/journal.pone.0094419

**Published:** 2014-04-10

**Authors:** Obiageli Crystal Oluka, Shaofa Nie, Yi Sun

**Affiliations:** 1 School of Public Health, Tongji Medical College, Huazhong University of Science and Technology, Wuhan, Hubei Province, China; Iran University of Medical Sciences, Iran (Islamic Republic Of)

## Abstract

**Objective:**

This review is aimed at assessing the quality of questionnaires and their development process based on the theory of planned behavior (TPB) change model.

**Methods:**

A systematic literature search for studies with the primary aim of TPB-based questionnaire development was conducted in relevant databases between 2002 and 2012 using selected search terms. Ten of 1,034 screened abstracts met the inclusion criteria and were assessed for methodological quality using two different appraisal tools: one for the overall methodological quality of each study and the other developed for the appraisal of the questionnaire content and development process. Both appraisal tools consisted of items regarding the likelihood of bias in each study and were eventually combined to give the overall quality score for each included study.

**Results:**

8 of the 10 included studies showed low risk of bias in the overall quality assessment of each study, while 9 of the studies were of high quality based on the quality appraisal of questionnaire content and development process.

**Conclusion:**

Quality appraisal of the questionnaires in the 10 reviewed studies was successfully conducted, highlighting the top problem areas (including: sample size estimation; inclusion of direct and indirect measures; and inclusion of questions on demographics) in the development of TPB-based questionnaires and the need for researchers to provide a more detailed account of their development process.

## Introduction

The Theory of Planned Behavior is a theoretical model of behavior change which proposes that behavior is best predicted by intention [1]. Intention in turn is dependent on: attitude (positive or negative) toward the behavior, subjective norms (social pressures to perform/not perform the proposed behavior) and perceived behavioral control (ability or difficulty of performing the behavior). The Theory of Planned Behavior (TPB) model is subjective in nature, inherently veered toward individualistic/personalized perception of human behavior. It implies that individuals will have the intention to perform a behavior when they evaluate it positively, believe that important others think they should perform it, and perceive it to be within their own control [2]. Measurement of intention requires measurement of its predictors which in the context of TPB is most commonly inferred from questionnaire responses.

Questionnaires are instruments used in research to gather valid and reliable information from respondents. It is an indispensable tool in behavior change studies as it helps in data collection for measurement of the constructs in various behavior models. To develop a good questionnaire, it is important that one not only understands the behavior change model and its constructs but also the specific behavior (in question) to which they will be applied. There are no standardized questionnaires for general measurement of TPB constructs for every behavior [3] rather, questions for each construct is developed based on the specific behavior of interest. Operationalization of the TPB model, therefore, requires definition of the specific health behavior of interest using the TACT principle (Target, Action, Context and Time) after which a questionnaire is tailored for it. Construction of these questionnaires are however, not as easy as it sounds.

Though TPB model has been proven to be effective and efficient in predicting health behavior based on a wide range of reviews [4], [5], none of these reviews have assessed the quality of the measurement tools employed in various studies. Also, most studies do not provide a detailed description of their questionnaire development process, thereby making it impossible to accurately assess their reliability. Data obtained from questionnaires should be reliable and as unbiased as possible, but are still subject to error and bias from a range of sources [6]. This may affect the efficiency of TPB model to predict health behavior. Guidelines for questionnaire development for TPB are described by Ajzen [7] and more recently by Francis J. et al. [3]. These guidelines have been cited in many studies and have probably reduced the difficulties encountered by researchers in the development process. However, to better ensure the validity and reliability of TPB-based questionnaires, it is necessary to assess their quality with the aid of a quality appraisal tool.

### Aims and Objectives

The aim of this review is to conduct a critical appraisal of TPB-based questionnaires and their development processes in order to provide a structured overview of its adequacy as a tool in predicting intention and behavior.

The objectives are to evaluate:

- How does the development process and content of TPB-based questionnaires influence the designing of effective behavior change interventions?
- What are the barriers to developing robust questionnaires?

## Methods

This review was based on studies that aimed at constructing a valid tool for measurement of the TPB constructs. It was conducted between March and May, 2013. A systematic literature search for published studies on TPB-based questionnaire construction for various health-related behaviors was conducted. Only studies published between the years 2002–2012 were utilized so as to obtain the most recent available evidence on the subject. The use of these recent studies was necessitated by the controversies surrounding the practicalization of TPB in the past, which has been somewhat resolved by the emergence of new evidence [3], [7], that has helped researchers better apply the theory. Key search terms were mapped out to generate as much evidence as possible. The inclusion criteria were expanded to include all health behavioral studies whose primary aim is to develop a questionnaire for predicting intention and/or behavior based on the proposed model. Focusing only on studies with the primary aim of questionnaire development is due to the fact that most TPB-based studies do not provide an explicit report of their questionnaire development process. Therefore, in order to avoid poor assessment of studies due to limited reporting of their questionnaire development processes, only studies with primary aim of developing questionnaires were included as they are expected to give a more detailed description of the development process.

It should be noted that this is a systematic review of evidence that aims at critically appraising the quality of questionnaires and its development process for various behavior studies based on TPB. However, no high level evidence studies (other systematic reviews, RCTs, etc.) were found in this subject area, limiting the included studies to only primary studies in the form of questionnaire surveys, focus groups and cross-sectional studies.

### Literature search

A computerized search for eligible articles was conducted using PUBMED, Cochrane Library, PsycINFO, PsycArticles and Google scholar from the year 2002–2012. In addition to electronic database searches, reference lists of eligible articles and National Institute for Health and Care Excellence (NICE) website were also searched. The search keywords were derived from synonyms of the following words; ‘behavior’, ‘intention’, ‘Theory of Planned Behavior’ and ‘questionnaire’, which were used in various combinations. Searches were limited to studies published in English language due to inadequate time and resources. A summary of the search strategy for each included database is provided in **Appendix S2 in** **File S1**.

### Inclusion and Exclusion criteria

Study selection was conducted based on the inclusion and exclusion criteria presented in **Table 1** below. Potentially relevant studies were identified by scanning their abstracts and titles. These were examined independently by two reviewers and an agreement reached on articles which did not meet the selection criteria. These two reviewers further independently examined the full texts of the remaining articles and they reached a consensus on those to be included.

**Table 1:** Inclusion and exclusion criteria.

Inclusion criteria	Exclusion criteria
Empirical articles in English	Studies not primarily aimed at instrument construction
Publication years from 2000 to 2012	Studies not reporting the questionnaire development process
Primary studies with primary aim of developing a questionnaire based on TPB	No report of content analysis of instrument
Focus on a particular behavior	Studies scoring <50% in quality assessment
Analysis of content validity and reliability of instrument	

### Quality Appraisal

Questionnaires are often used to collect primary quantitative data from patients and healthcare professionals; however, most behavioral studies that report the development of TBP-based questionnaires are qualitative in nature [6], [8]. Therefore, a quality appraisal tool for qualitative research (**Table 2**), adapted from Appendix H of the NICE ‘Methods for the development of NICE public health guidance manual’ [9] was used to assess the quality of the individual selected studies. The quality of each study was graded based on the criteria presented in the table with scores of ++ (low level of bias); + (moderate level of bias); or – (variable quality with higher degree of bias). The criteria was considered to be unfulfilled if (a) it was clearly stated, (b) a characteristic was not described, or (c) not enough detail was provided to make a decision.

**Table 2:** Criteria used to assess study quality.

Criteria	Score
1. Clear aim or research question?	+/−
2. Details of study methodology and design?	+/−
3. Description of data collection?	+/−
4. Research context; description of the study population?	+/−
5. Data analysis?	+/−
6. Results relevant to the aims of the study?	+/−
7. Ethical approval obtained?	+/−
Overall Assessment	++/+/−

Notes:

++ must meet at least 6 criterion indicated above.

+ must meet at least 4 criterion indicated above.

− did not have the 4 criterion necessary for + classification.

++ denotes low level of bias; + denotes moderate level of bias; and – denotes higher degree of bias.

Since the primary focus of this study is to assess the quality of questionnaire construction, a quality assessment checklist (**Table 3**) was developed with guidance from ‘Construction of questionnaires based on the theory of planned behavior manual for health service researchers’ [3]. The checklist was based on the potential for bias and evaluated using score points; those meeting the quality standard were given a score of one, while those not meeting the standard were given a score of zero. A criterion for number of reviewers or experts involved in choosing of items for the questionnaire was included as at least two reviewers are required to reduce the risk of selection bias. Other criteria are based on recommendations from the manual including: inclusion of direct and indirect measures for which it is recommended that neither approach is perfect so both should be included. Determination of sample size by statistical power analysis was also included as a criterion; however, a sample size of 80 is generally deemed acceptable [3]. Scoring of items, choice of scaling and response formats are not included as part of the criteria due to the weight of controversies around them [10]. Questions on elicitation study and total number of items in the questionnaire, though included in the checklist, were excluded in the scoring, as some studies may have adopted an already existing instrument and depending on their aim, use only a brief form of the questionnaire. Studies that adopt an existing instrument would not require an elicitation study while those whose aim allow for a brief questionnaire format, will have the total number of their questionnaire items reduced. Therefore, three items on the elicitation study (B 3, 4 & 5) and one (C 10) on number of questionnaire items were excluded from the overall scoring, leaving a total of 12 criteria. Scoring of studies was adopted from Jack *et al*. and Husebo *et al*., and then modified to fit the number of items in our assessment tool [11], [12]. Studies scoring ≥7 (above average) were considered ‘high quality’ (Grade A) while those <7 were rated ‘low quality’ (Grade B). Any disagreements on quality criteria and scoring were resolved by consensus.

**Table 3:** Quality appraisal criteria for questionnaire development.

Criteria		Score
A	1. Definition of population of interest?	+/−/?
	2. Definition of clinical condition and/or behavior of Interest (TACT principle)?	+/−/?
B	3. Elicitation study conducted for salient beliefs? If yes,	+/−/?
	4. Study design/mode of administration stated (focus groups/individual or mailed questions)?	+/−/?
	5. Are participants from target population?	+/−/?
C	6. Is more than one reviewer/expert involved in choosing of questionnaire items?	+/−/?
	7. Are all the constructs represented?	+/−/?
	8. Inclusion of direct and indirect measures?	+/−/?
	9. Inclusion of questions on demographics?	+/−/?
	10. Total number of questionnaire items ≥40?	+/−/?
D	11. Was a pilot study conducted?	+/−/?
E	12. Was power calculation done for final study?	+/−/?
	13. Total number of participants' ≥80?	+/−/?
	14. Is sample representative of study population (higher response rate or characteristics compared between responders and non-responders)?	+/−/?
F	15. Ethical approval obtained?	+/−/?
G	16. Data analysis conducted for content validity/reliability?	+/−/?

**Notes**: Studies scoring ≥7 were considered ‘high quality’ (Grade A) while those <7 were rated ‘low quality’ (Grade B). Scoring adopted from Husebo *et al*. (2012) and Jack *et al*. (2010).

Overall quality score was provided based on the classification of bias (e.g. ++) of the individual studies and that of the questionnaire assessment (e.g. Grade A).

### Data extraction and synthesis

Data from relevant studies were abstracted on the methodological quality of the studies, characteristics of study setting, participants, targeted behaviors, predictor variables, data analysis and results. These were extracted directly into templates modified from a version provided in the Center for Public Health Education (CPHE) methods manual [9], providing similarities and differences between the studies. Results are presented in form of narrative summaries with further information on each study provided in evidence tables adapted from Appendix K of the NICE ‘Methods for the development of NICE public health guidance manual’.

## Results

A total of 10 studies were included in this review and their common characteristics are presented in evidence tables. Initial data search using a combination of the aforementioned search terms yielded 1,052 titles possibly relevant to the research question. Titles and abstracts were further screened and irrelevant ones discarded. Full texts of the remaining 28 abstracts were retrieved and examined in detail by two reviewers against the inclusion and exclusion criteria presented in Table 1, narrowing it down to 10 potential articles for inclusion. One study [13], whose primary aim was to only present the content validity and reliability of an instrument was almost excluded but upon further debate was considered for inclusion as its aim is still a part of the questionnaire development process. All the included studies were published between 2005 and 2012. A flow diagram of the search is included in **Appendix S1 in** **File S1**.

### Characteristics of reviewed studies

The main characteristics of the reviewed studies are presented as part of the data in the evidence tables (**Tables 4** **,** **5** **,** **6** **,** **7**). Four of the studies were based on physical activity/exercise in different populations [14], [15], [16], [17]; two were based on intention to seek mammography [18], [19]; one measured adolescents attitudes towards breast feeding [20]; one assessed blood donation behavior among university students [21]; one was based on HIV/AIDS behavior surveillance [22]; and one was on determinants of salt intake among the hypertensive [13]. The different behaviors listed, were assessed using different study designs and in different populations in terms of age, sex, geography, ethnicity and other demographic information. Also, all the reviewed studies were primarily aimed at questionnaire development except for one [13] which was aimed at only presenting the content validity and reliability of an instrument. Data analysis for each included study had significant results for content validity and reliability, with the later based on internal consistency assessed by test-retest reliability (for indirect measures) and/or Cronbach's alpha (for direct measures). The reliability results were all >0.60 as recommended by Francis et al [3].

**Table 4:** Evidence table.

Study	Theoretical framework	Population	Design	Outcome	Predictive factors	Quality score (overall)	Results
Lopez-Mckee (2010)	Theory of planned behavior (TPB)	N = 14; Women; Mean age = 58 years; Low-health-literacy Mexican American women	Focus group study	Intention to seek mammography screening	TPB constructs	8++	Development of an instrument to measure cultural and psychosocial behavioral factors impacting intention to seek mammography screening. Significant content validity
Steele & Porche (2005)	TPB	N = 302; Women; Mean age = 57 years; No history of breast cancer; Rural Southeastern Louisiana	Questionnaire survey	Intention to seek mammography	TPB constructs; Accessible beliefs	10++	Development of a questionnaire to predict intention to seek mammography. Significant content reliability and validity

**Table 5:** Evidence table.

Study	Theoretical framework	Population	Design	Outcome	Predictive factors	Quality score (overall)	Results
Gonzalez et al. (2012)	TPB	N = 135; Both sexes; Mean age = 27 years; General population in Spain	Questionnaire survey	Practicing physical exercise	TPB constructs	8+	Development of an instrument assessing TPB constructs in the context of practicing physical exercise. Significant content reliability and validity
Giles et al. (2007)	TPB	N = 121; Both sexes; Mean age = 13–14 years; Adolescents in Northern Ireland	Focus groups/Questionnaire survey	Attitude towards breastfeeding	TPB constructs; Accessible beliefs; Self-efficacy; Knowledge	8++	Development of an instrument for subsequent use in large scale attitude survey
Jalalian et al. (2010)	TPB	N = 24; Both sexes; Mean age = −; Students at a University in Malaysia	Cross-sectional pilot study	Intention to donate blood	TPB constructs; Self-efficacy; Accessible beliefs; Knowledge	8++	Development of a measurement tool for assessing blood donation behavior. Adequate content reliability

**Table 6:** Evidence table.

Study	Theoretical framework	Population	Design	Outcome	Predictive factors	Quality score (overall)	Results
Ana et al. (2012)	TPB	N = 90; Women; Mean age = 56–58 years; Breast cancer survivors; Prior surgical treatment Puerto Rico	Questionnaire survey	Intention to exercise among breast cancer survivors	TPB constructs; Accessible beliefs	8++	Development of a questionnaire to assess the determinants of exercise. Significant content validity and reliability
Ghazanfari et al. (2010)	TPB	N = 127; Women; Mean age = 46.40 years; Diabetics with no history of complications in Tehran, Iran	Cross-sectional study	Beliefs about physical activity	TPB constructs; Self-identity	10++	Development of an instrument (PAQ-DP) for assessing women's perceptions about physical activity. Significant content validity and reliability

**Table 7:** Evidence table.

Study	Theoretical framework	Population	Design	Outcome	Predictive factors	Quality score (overall)	Results
Boileau et al. (2008)	TPB	N = 531; Both sexes; Mean age = 15–24 years; In Bamako, Mali	Focus group/Questionnaire survey	Surveillance of HIV risk behavior	TPB constructs; Knowledge Communication	9++	Development of a comprehensive instrument for behavior surveillance of HIV/AIDS and STIs in urban West Africa. Significant content validity and reliability
Mendez et al. (2010)	TPB	N = 51; Both sexes; Mean age = −; Patients with coronary disease at a clinic in Sao Paulo	Questionnaire survey	Physical activity behavior (walking 3 times a week for at least 30 minutes)	TPB constructs; Self-efficacy; Habit; Perceived risk	7++	Development of a questionnaire for determining the psychosocial factors of physical activity behavior. Significant content validity and reliability
Cornelio et al. (2009)	TPB	N = 32; Both sexes; Mean age = 55.13 years; Hypertension diagnosis	Questionnaire survey	Salt consumption	TPB constructs; Self-efficacy; Habit; Environment; Food preferences; Hedonic determinants	6+	Significant content validity and reliability of an instrument to measure determinants of salt consumption among hypertensive subjects

### Methodological quality

The quality appraisal results for the individual included studies are summarized in **Table 8** below. It shows that approximately 8 of the studies have a low risk of bias in all the domains of risk assessment with an overall assessment value of ++. Two of the included studies [13], [14], show a moderate risk of bias (+). The most common sources of potential methodological bias are related to description of data collection, data analysis and ethical issues.

**Table 8:** Results of Study Quality Appraisal.

Study	1	2	3	4	5	6	7	Quality Score
Lopez-Mckee (2010)	+	+	+	+	+	+	+	++
Steele & Porche (2005)	+	−	+	+	+	+	+	++
Gonzalez et al. (2012)	+	−	+	+	+	+	−	+
Giles et al. (2007)	+	+	+	+	+	+	−	++
Jalalian et al. (2010)	+	+	+	+	+	+	+	++
Ana et al. (2012)	+	−	+	+	+	+	+	++
Ghazanfari et al. (2010)	+	+	+	+	+	+	+	++
Boileau et al. (2008)	+	+	+	+	+	+	−	++
Mendez et al. (2010)	+	−	+	+	+	+	+	++
Cornelio et al. (2009)	+	−	−	+	+	+	+	+

Quality appraisal of the questionnaire construction shown in **Table 9** below yielded scores ranging from 6 to 10 indicating that all studies are of high quality (Grade A) except one. However, it is noteworthy that the study with the low quality score of 6 [13] was aimed at only assessing the content validity and reliability of an instrument and so, did not provide a detailed description of the instrument development process. Most methodological shortfalls are related to power calculation (in all 10 studies), use of direct and indirect measures (5 studies), demographic questions (5 studies) and representativeness of the sample (5 studies).

**Table 9:** Results of questionnaire quality appraisal.

Study	1	2	3	4	5	6	7	8	9	10	11	12	13	14	15	16	Quality Score
Lopez-Mckee (2010)	1	1	-	-	-	1	1	0	1	-	1	0	0	0	1	1	8
Steele & Porche (2005)	1	1	-	-	-	0	1	1	1	-	1	0	1	1	1	1	10
Gonzalez et al. (2012)	1	1	-	-	-	1	1	0	0	-	1	0	1	1	0	1	8
Giles et al. (2007)	1	1	-	-	-	0	1	1	0	-	1	0	1	1	0	1	8
Jalalian et al. (2010)	1	1	-	-	-	0	1	1	1	-	1	0	0	0	1	1	8
Ana et al. (2012)	1	1	-	-	-	1	1	1	0	-	1	0	0	0	1	1	8
Ghazanfari et al. (2010)	1	1	-	-	-	1	1	0	1	-	1	0	1	1	1	1	10
Boileau et al. (2008)	1	1	-	-	-	1	1	0	1	-	1	0	1	1	0	1	9
Mendez et al. (2010)	1	1	-	-	-	1	1	0	0	-	1	0	0	0	1	1	7
Cornelio et al. (2009)	0	1	-	-	-	1	1	0	0	-	1	0	0	0	1	1	6

## Discussion

The importance of behavior change theories in the fields of behavioral medicine/sciences and public health as a whole cannot be over-emphasized. To adequately substantiate an intervention that ultimately seeks to change health behavior, behavior change models that employ questionnaires as a measurement tool, require that valid and reliable questionnaires be used. This review is a critical appraisal of questionnaires developed for a variety of behaviors, in different populations, based on TPB. It provides a reasonable overview of the quality issues encountered in the development of an adequate measurement tool for predicting behavior based on the model of interest. The quality appraisal results of the 10 included studies showed evidence of low (++) to moderate (+) potential for bias. The checklist constructed for quality appraisal of the questionnaires also showed evidence of low potential for bias in 8 of the studies. There are, however, no previous studies that assessed questionnaire quality based on TPB that the results obtained in this review can be compared to. This may constitute strength as well as a limitation.

Bearing in mind that the sample size of a primary study, will ultimately influence inferences made on the general population of these studies, this criterion was included in the questionnaire quality assessment tool along with the criterion for power analysis. Sample size estimation is dependent on study design and the expected effect size [23]. Most of the study designs stated in this review were inferred and not categorically stated in the included studies, and none of the studies reported conducting a power analysis for determining their sample size. This makes it difficult to determine if their sample sizes are appropriate. Regardless, all included studies were assessed on these criteria based on the recommendation of Francis J. et al. [3] that a sample size of 25 and 80 participants are acceptable for the elicitation/pilot and final studies, respectively. The authors however, stated that these values are just recommendations and could be legitimately adjusted depending on the principles of the research such as sampling until data saturation is achieved. On that note, included studies with sample size less than 80 [16], [18], [21] may have been misjudged and also, they are all pilot studies except for one (Cornelio et al. [13]), which conducted its main/final study on only 32 participants.

The content reliability analysis in most of the included studies was conducted using Cronbach's alpha and was considered for meta-analysis in the four studies on physical exercise [14]–[17]. However, though the outcomes are the same, a meta-analysis was thought to be infeasible as the studies were considered too clinically diverse [24].

Theoretical and research literature surrounding the TPB is often confusing as it contains diverse views on how to operationalize the theory [3]. Researchers are forced to employ different views in their studies as they see fit. This has made it impossible to develop a more comprehensive critical appraisal tool (including items such as: scoring and response formats) for quality assessment of the questionnaires in the included studies. It may also limit the generalisability of the developed tool.

Some of the limitations encountered in this review are none inclusion of TPB-based studies whose primary aim is not questionnaire development, despite the fact that they may have provided a detailed report of their questionnaire development process. Moreover, the limited number of included studies, prompted by the fact that most TPB-based studies do not elaborate on their questionnaire development process; may have reduced the validity and generalisability of the conclusions drawn in this review. Relevant studies in databases not assessed in this review may have been omitted. Unpublished studies and non-English language papers not included may also constitute a limitation.

### How does the development process and content of TPB-based questionnaires influence the designing of effective behavior change interventions?

Questionnaires have proven to be a reliable and indispensable tool in belief elicitation as is evidenced by the vast volume of research that have indicated the effectiveness of TPB in predicting health behaviors and effecting behavioral change [4], [5]. From the elicitation of salient beliefs to the wording and formatting of items, the role of questionnaires in predicting behavior towards designing an effective intervention is undebatable. The 10 studies included in this review started their process of behavior prediction with an elaborate questionnaire construction process, highlighting the link between questionnaires and the planning of effective interventions. The study on breastfeeding [20] associated breastfeeding intention to salient beliefs of providing health benefits for the baby and mother as well as limiting social activity and causing embarrassment. These beliefs were used to construct questionnaires for the target population with the aim of eventually developing an effective intervention for breastfeeding. The four studies on physical exercise [14]–[17] and the other included studies all successfully employed questionnaires in assessing salient beliefs of different populations with the aim of applying their results in clinical practice. It is difficult to conceive of another method more appropriate since the structured nature of questionnaires as well as their contents may yield data more comparable than information obtained from other forms of interview [25], [26]. Development of a valid and reliable questionnaire is a vital step in achieving an effective intervention.

### What are the barriers to developing robust questionnaires?

Robust or sound questionnaires may be described as those which are content valid, reliable and able to correctly measure target behavior. For the purpose of this study, possible barriers in development of robust questionnaires are mostly related to potential sources of bias. Most of these sources of bias are included in the questionnaire critical appraisal tool in Table 3. Others not included are:

- **Complexity of questionnaire items**: this encompasses the wording of questions which may be confusing for the respondents (e.g. as seen in the Mammography screening study by Lopez-Mckee) [18] or may not be right for measuring the target behavior. It also includes the choice of endpoints and response format employed though this is still a controversial area [27].
- **Length of the questionnaire**: It is reported that most respondents in various studies think TPB questionnaires are rather long [20]. Regardless, the length of these questionnaires should not be taken for granted as it can reduce response or completion rates due to decreased motivation.
- **Extent to which the respondent believes his/her responses are important**: this would likely occur when respondents are not adequately informed of the study aims. This can be avoided by providing information sheets to the respondents prior to questionnaire administration.

## Conclusion

There are promises and pitfalls in theory based research. Though the TPB has been proven reliable by a vast evidence base, studies based on the theory can produce invalid and unreliable evidence if questionnaire quality is low. Quality appraisal of the questionnaires in the 10 reviewed studies was successfully conducted. There is, however, still need for a more comprehensive and standardized appraisal tool. It is recommended that research first be conducted to resolve the controversies in the operationalization of the TPB model, and afterward develop a standardized checklist for assessing the quality of its questionnaires. Also, researchers using the TPB model should provide a more detailed account of their questionnaire development process.

## Supporting Information

Checklist S1 **Prisma Checklist.**
(DOCX)

File S1 **Appendix S1.** Flowchart of study search. Of 1052 records identified, 1042 were excluded and 10 studies selected for inclusion. **Appendix S2.** Details of Literature Search. Systematic search of selected databases with 6986 references found and 1052 retrieved as possibly relevant. **Appendix S3.** Sample of NICE Checklist for Study Quality Assessment. A comprehensive checklist of study quality assessment with overall assessment scores of ++/+/−. **Appendix S4.** List of some Excluded studies.
(DOC)
