# Supplementary material for: Quality Assessment of TPB-Based Questionnaires: A Systematic Review
Source: PLoS One. 2014 Apr 10;9(4):e94419. doi: 10.1371/journal.pone.0094419 (PMC3983195; doi:10.1371/journal.pone.0094419)
Supplement: File S1 — Appendix S1. Flowchart of study search. Of 1052 records identified, 1042 were excluded and 10 studies selected for inclusion. Appendix S2. Details of Literature Search. Systematic search of selected databases with 6986 references found and 1052 retrieved as possibly relevant. Appendix S3. Sample of NICE Checklist for Study Quality Assessment. A comprehensive checklist of study quality assessment with overall assessment scores of ++/+/−. Appendix S4. List of some Excluded studies. (DOC) [file pone.0094419.s002.doc]

**Appendix S1: Flowchart of study search**

Records identified (N= 1,052)

Pubmed (n= 904)

Cochrane Library (n= 8)

Google scholar (n = 122)

PsycINFO (n = 15)

PsycArticles (n = 3)

1,024 excluded (based on further screening of abstracts and titles)

Full texts assessed for eligibility

N= 28

Excluded studies (N = 18)

Different aim (n= 8)

Different publication year (n = 5)

Not published in English (n = 2)

No analysis of content validity and reliability (n = 3)

Studies selected for inclusion

N= 10

**Appendix S2**: Details of Literature Search

| **Database Name** | **References found** | **References retrieved** (*based on preliminary screening of titles and abstracts*) |
| --- | --- | --- |
| Pubmed | 1799 | 904 |
| Cochrane Library | 79 | 8 |
| PsycINFO | 2961 | 15 |
| Google Scholar | 2036 | 122 |
| PsycArticles | 111 | 3 |
| Total | 6986 | 1052 |

**Appendix S3**: Sample of NICE Checklist for Study Quality Assessment.

| **Study identification:** Include author, title, reference, year of publication |  | |
| --- | --- | --- |
| **Guidance topic:** | **Key research question/aim:** | |
| **Checklist completed by:** |  | |
| **Theoretical approach** | | |
| **1. Is a qualitative approach appropriate?**  For example:   - Does the research question seek to understand processes or structures, or illuminate subjective experiences or meanings? - Could a quantitative approach better have addressed the research question? | Appropriate  Inappropriate  Not sure | Comments: |
| **2. Is the study clear in what it seeks to do?**  For example:   - Is the purpose of the study discussed – aims/objectives/research question/s? - Is there adequate/appropriate reference to the literature? - Are underpinning values/assumptions/theory discussed? | Clear  Unclear  Mixed | Comments: |
| **Study design** | | |
| **3. How defensible/rigorous is the research design/methodology?**  For example:   - Is the design appropriate to the research question? - Is a rationale given for using a qualitative approach? - Are there clear accounts of the rationale/justification for the sampling, data collection and data analysis techniques used? - Is the selection of cases/sampling strategy theoretically justified? | Defensible  Indefensible  Not sure | Comments: |
| **Data collection** | | |
| **4. How well was the data collection carried out?**  For example:   - Are the data collection methods clearly described? - Were the appropriate data collected to address the research question? - Was the data collection and record keeping systematic? | Appropriately  Inappropriately  Not sure/inadequately reported | Comments: |
| **Trustworthiness** | | |
| **5. Is the role of the researcher clearly described?**  For example:   - Has the relationship between the researcher and the participants been adequately considered? - Does the paper describe how the research was explained and presented to the participants? | Clearly described  Unclear  Not described | Comments: |
| **6. Is the context clearly described?**  For example:   - Are the characteristics of the participants and settings clearly defined? - Were observations made in a sufficient variety of circumstances - Was context bias considered | Clear  Unclear  Not sure | Comments: |
| **7. Were the methods reliable?**  For example:   - Was data collected by more than 1 method? - Is there justification for triangulation, or for not triangulating? - Do the methods investigate what they claim to? | Reliable  Unreliable  Not sure | Comments: |
| **Analysis** | | |
| **8. Is the data analysis sufficiently rigorous?**  For example:   - Is the procedure explicit – i.e. is it clear how the data was analysed to arrive at the results? - How systematic is the analysis, is the procedure reliable/dependable? - Is it clear how the themes and concepts were derived from the data? | Rigorous  Not rigorous  Not sure/not reported | Comments: |
| **9. Is the data 'rich'?**  For example:   - How well are the contexts of the data described? - Has the diversity of perspective and content been explored? - How well has the detail and depth been demonstrated? - Are responses compared and contrasted across groups/sites? | Rich  Poor  Not sure/not reported | Comments: |
| **10. Is the analysis reliable?**  For example:   - Did more than 1 researcher theme and code transcripts/data? - If so, how were differences resolved? - Did participants feed back on the transcripts/data if possible and relevant? - Were negative/discrepant results addressed or ignored? | Reliable  Unreliable  Not sure/not reported | Comments: |
| **11. Are the findings convincing?**  For example:   - Are the findings clearly presented? - Are the findings internally coherent? - Are extracts from the original data included? - Are the data appropriately referenced? - Is the reporting clear and coherent? | Convincing  Not convincing  Not sure | Comments: |
| **12. Are the findings relevant to the aims of the study?** | Relevant  Irrelevant  Partially relevant | Comments: |
| **13. Conclusions**  For example:   - How clear are the links between data, interpretation and conclusions? - Are the conclusions plausible and coherent? - Have alternative explanations been explored and discounted? - Does this enhance understanding of the research topic? - Are the implications of the research clearly defined?   **Is there adequate discussion of any limitations encountered?** | Adequate  Inadequate  Not sure | Comments: |
| **Ethics** | | |
| **14. How clear and coherent is the reporting of ethics?**  For example:   - Have ethical issues been taken into consideration? - Are they adequately discussed e.g. do they address consent and anonymity? - Have the consequences of the research been considered i.e. raising expectations, changing behaviour? - Was the study approved by an ethics committee? | Appropriate  Inappropriate  Not sure/not reported | Comments: |
| **Overall assessment** | | |
| **As far as can be ascertained from the paper, how well was the study conducted? (see guidance notes)** | ++  +  − | Comments: |

**++** All or most of the checklist criteria have been fulfilled; where they have not been fulfilled the conclusions are very unlikely to alter**. +** Some of the checklist criteria have been fulfilled, where they have not been fulfilled, or not adequately described, the conclusions are unlikely to alter. **–** Few or no checklist criteria have been fulfilled and the conclusions are likely or very likely to alter.

**Appendix S4: List of some Excluded studies**

1. [Barnes AS](http://www.ncbi.nlm.nih.gov/pubmed?term=Barnes AS%5BAuthor%5D&cauthor=true&cauthor_uid=17415617), [Goodrick GK](http://www.ncbi.nlm.nih.gov/pubmed?term=Goodrick GK%5BAuthor%5D&cauthor=true&cauthor_uid=17415617), [Pavlik V](http://www.ncbi.nlm.nih.gov/pubmed?term=Pavlik V%5BAuthor%5D&cauthor=true&cauthor_uid=17415617), [Markesino J](http://www.ncbi.nlm.nih.gov/pubmed?term=Markesino J%5BAuthor%5D&cauthor=true&cauthor_uid=17415617), [Laws DY](http://www.ncbi.nlm.nih.gov/pubmed?term=Laws DY%5BAuthor%5D&cauthor=true&cauthor_uid=17415617), [Taylor WC](http://www.ncbi.nlm.nih.gov/pubmed?term=Taylor WC%5BAuthor%5D&cauthor=true&cauthor_uid=17415617). (2007). Weight Loss Maintenance in African–American Women: Focus Group Results and Questionnaire Development. *Society of General internal Medicine;* 22: 915-922.
2. Eun-Seok C, Kevin HK, Thelma E. (2008). Patrick. Predictors of Intention to Practice Safer Sex Among Korean College Students. *Arch Sex Behav*; 37:641–651.
3. Nada O. Kassem, Jerry W. Lee, Naomi N. Modeste and Patricia K. Johnston. (2003). Understanding soft drink consumption among female adolescents using the Theory of Planned Behavior. *Health Education Research Theory and Practise*; 18 (3): 278-291.
4. Wade J., Smith H., Hankins M., Llewellyn C. (2009). Conducting oral examinations for cancer in general practice: what are the barriers? *Fam Pract*; 27: 77-84.
5. Prior M, Burr JM., Ramsay CR., Jenkinson D, Campbell S, Francis JJ. (2012). Evidence base for an intervention to maximize uptake of glaucoma testing: a theory-based cross-sectional survey. *BMJ Open*; 2: e000710.
6. Hardeman W, Prevost AT, Parker RA, Sutton S. (2013). Constructing multiplicative measures of beliefs in the theory of planned behavior. *Br J Health Psychol;* 18: 122-138.
7. Hardeman W, Johnston M, Johnston DW, Bonetti D, Wareham NJ, Kinmonth AL. (2002). Application of the Theory of Planned Behavior in behavior change interventions: A systematic review. *Psychol Health*; 17 (2): 123-158.

**AMSTAR Checklist**

| **1. Was an 'a priori' design provided?** The research question and inclusion criteria should be established before the conduct of the review. | No |
| --- | --- |
| **2. Was there duplicate study selection and data extraction?** There should be at least two independent data extractors and a consensus procedure for disagreements should be in place. | Yes |
| **3. Was a comprehensive literature search performed?** At least two electronic sources should be searched. The report must include years and databases used (e.g., Central, EMBASE, and MEDLINE). Key words and/or MESH terms must be stated and where feasible the search strategy should be provided. All searches should be supplemented by consulting current contents, reviews, textbooks, specialized registers, or experts in the particular field of study, and by reviewing the references in the studies found. | Yes |
| **4. Was the status of publication (i.e. grey literature) used as an inclusion criterion?** The authors should state that they searched for reports regardless of their publication type. The authors should state whether or not they excluded any reports (from the systematic review), based on their publication status, language etc. | Yes |
| **5. Was a list of studies (included and excluded) provided?** A list of included and excluded studies should be provided. | Yes |
| **6. Were the characteristics of the included studies provided?** In an aggregated form such as a table, data from the original studies should be provided on the participants, interventions and outcomes. The ranges of characteristics in all the studies analyzed e.g., age, race, sex, relevant socioeconomic data, disease status, duration, severity, or other diseases should be reported. | Yes |
| **6. Were the characteristics of the included studies provided?** In an aggregated form such as a table, data from the original studies should be provided on the participants, interventions and outcomes. The ranges of characteristics in all the studies analyzed e.g., age, race, sex, relevant socioeconomic data, disease status, duration, severity, or other diseases should be reported. | Yes |
| **7. Was the scientific quality of the included studies assessed and documented?** 'A priori' methods of assessment should be provided (e.g., for effectiveness studies if the author(s) chose to include only randomized, double-blind, placebo controlled studies, or allocation concealment as inclusion criteria); for other types of studies alternative items will be relevant. | Yes |
| **8. Was the scientific quality of the included studies used appropriately in formulating conclusions?** The results of the methodological rigor and scientific quality should be considered in the analysis and the conclusions of the review, and explicitly stated in formulating recommendations. | Yes |
| **9. Were the methods used to combine the findings of studies appropriate?** For the pooled results, a test should be done to ensure the studies were combinable, to assess their homogeneity (i.e., Chi-squared test for homogeneity, I2). If heterogeneity exists a random effects model should be used and/or the clinical appropriateness of combining should be taken into consideration (i.e., is it sensible to combine?). | Not applicable |
| **10. Was the likelihood of publication bias assessed?** An assessment of publication bias should include a combination of graphical aids (e.g., funnel plot, other available tests) and/or statistical tests (e.g., Egger regression test, Hedges-Olken). | No |
| **11. Was the conflict of interest included?** Potential sources of support should be clearly acknowledged in both the systematic review and the included studies. | No |
